# Temporal-to-Nasal Macular Ganglion Cell and Inner Plexiform Layer Ratios in a Large Adult Twin Cohort: Correlations With Age and Heritability

**DOI:** 10.1167/iovs.65.2.26

**Published:** 2024-02-13

**Authors:** Zakariya A. Jarrar, Khaldoon O. Al-Nosairy, Xiaofan Jiang, Ali Lamin, Dominic Wong, Abdus S. Ansari, Katie M. Williams, Sobha Sivaprasad, Michael B. Hoffmann, Pirro G. Hysi, Christopher J. Hammond, Omar A. Mahroo

**Affiliations:** 1 Section of Ophthalmology, King's College London, St. Thomas’ Hospital Campus, London, United Kingdom; 2 Department of Twin Research and Genetic Epidemiology, King's College London, London, United Kingdom; 3 Department of Ophthalmology, Faculty of Medicine, Otto-von-Guericke University, Magdeburg, Germany; 4 NIHR Biomedical Research Centre at Moorfields Eye Hospital and the UCL Institute of Ophthalmology, London, United Kingdom; 5 Institute of Ophthalmology, University College London, London, United Kingdom; 6 Center for Behavioral Brain Sciences, Magdeburg, Sachsen-Anhalt, Germany

**Keywords:** retina, retinal ganglion cells, optical coherence tomography

## Abstract

**Purpose:**

Temporal-to-nasal macular ganglion cell layer thickness ratios are reduced in albinism. We explored similar ratios in a large twin cohort to investigate ranges in healthy adults, correlations with age, and heritability.

**Methods:**

More than 1000 twin pairs from TwinsUK underwent macular optical coherence tomography (OCT) scans. Automated segmentation yielded thicknesses for the combined ganglion cell and inner plexiform layer (GCIPL) in Early Treatment of Diabetic Retinopathy Study subfields. Participants with diseases likely to affect these layers or segmentation accuracy were excluded. Inner and outer ratios were defined as the ratio of temporal-to-nasal GCIPL thickness for inner and outer subfields respectively. Corresponding ratios were obtained from a smaller cohort undergoing OCTs with a different device (three-dimensional (3D)-OCT, Topcon, Japan).

**Results:**

Scans from 2300 twins (1150 pairs) were included (mean [SD] age, 53.9 (16.5) years). Mean (SD) inner and outer ratios were 0.89 (0.09) and 0.84 (0.11), correlating negatively with age (coefficients, −0.17 and −0.21, respectively). In males (150 pairs) ratios were higher and did not correlate significantly with age. Intrapair correlation coefficients were higher in monozygotic than dizygotic pairs; age-adjusted heritability estimates were 0.20 and 0.23 for inner and outer ratios, respectively. For the second cohort (n = 166), mean (SD) ratios were 0.93 (0.08) and 0.91 (0.09), significantly greater than for the larger cohort.

**Conclusions:**

Our study gives reference values for temporal-to-nasal macular GCIPL subfield ratios. Weak negative correlations with age emerged. Genetic factors may contribute to ∼20% to 23% of the variance in healthy individuals. The ratios differ according to the OCT platform used.

Retinal ganglion cells are selectively affected in several clinical conditions, and their spatial distribution is also relevant. Ganglion cells are usually absent from the foveal center, whereas patients with foveal hypoplasia, often associated with ocular or oculocutaneous albinism, have persistence of inner retinal layers in this region. Segmented macular layer thicknesses, obtained from optical coherence tomography (OCT) scans, are conventionally partitioned according to Early Treatment of Diabetic Retinopathy Study (ETDRS) subfields.^1^^–^^6^ The ratio of temporal to nasal macular ETDRS subfield ganglion cell layer thickness has been shown to be reduced in albinism compared with the ratio in healthy individuals,^7^^–^^9^ and this can aid in diagnosis, for example, in the investigation of nystagmus. Such ratios have been found to be reduced in albinism and not in other causes of nystagmus.^9^

Exploring the range for these ratios in the healthy population is of interest. As well as helping define what might be considered a clinically relevant abnormality, such studies can yield insight into the spectrum of healthy foveal development and changes with age. Also, twin studies enable an estimation of the extent to which genetic factors contribute to the variance in phenotypic parameters in the general population. In this study, we obtained thickness data for the combined ganglion cell and inner plexiform layer (GCIPL), and quantified the temporal-to-nasal GCIPL ratios in over 1000 adult twin pairs, investigating correlations with age and coefficients of intrapair correlation in monozygotic (MZ) and dizygotic (DZ) twin pairs, as well as formally estimating heritability. We also examined data from a smaller cohort of 176 twins in whom OCT scans had been obtained with a different platform to explore whether measurements were comparable and whether broadly similar changes with age were observed.

## Methods

### Participants

Individuals were recruited from the TwinsUK registry,^10^ a UK-based cohort of adult twins who have volunteered to participate in research studies based at St. Thomas’ Hospital in London. Participants gave informed consent. The study had local research ethics committee approval and was conducted in accordance with the tenets of the Declaration of Helsinki. All twin pairs in this study were same-sex. When the twins were first recruited to the registry, for each recruited twin pair, the first recruited twin was designated “Twin 1” and the fellow twin designated “Twin 2.”

### Imaging and Segmentation

**Figure 1.**
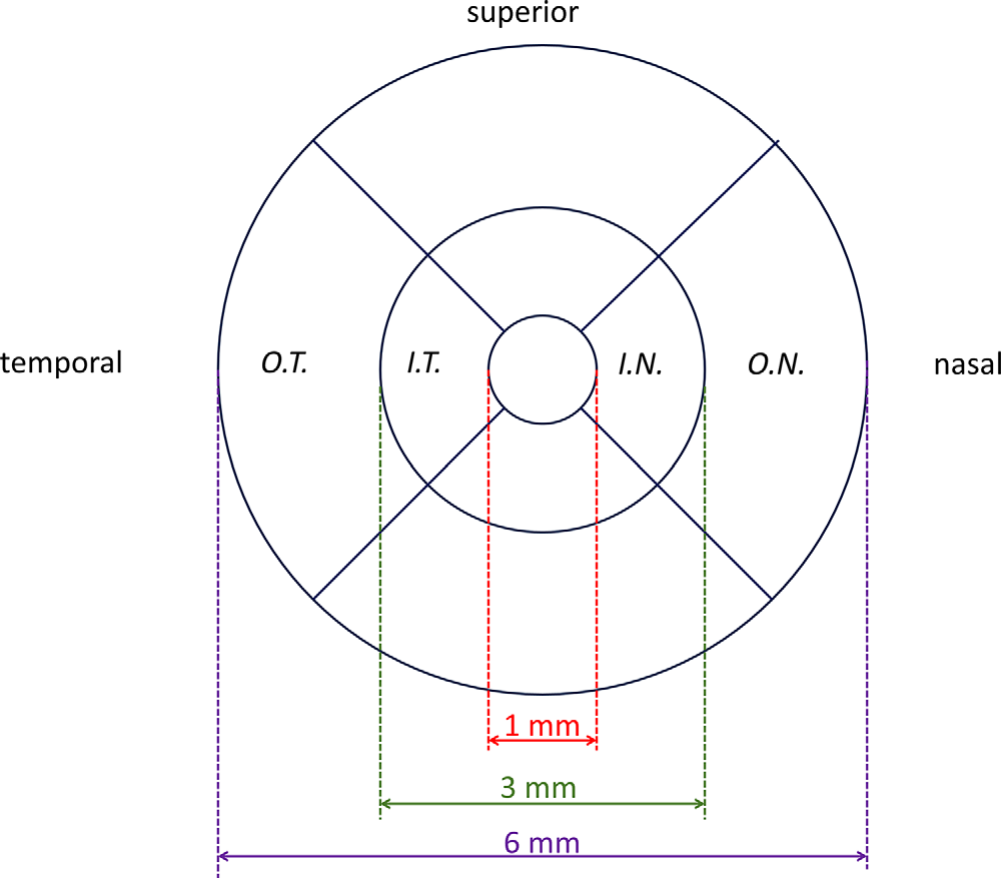
Schematic showing ETDRS subfields relevant to the present study. The fields shown are for the right eye and the circles are centered on the foveal center. OT, IT, IN, and ON refer to the outer temporal, inner temporal, and inner nasal and outer nasal subfields, respectively. The “inner ratio” is calculated by dividing the GCIPL thickness for the IT subfield by the corresponding thickness for the IN subfield. The “outer ratio” is calculated by dividing the GCIPL thickness for the OT subfield by the corresponding thickness for the ON subfield.

Macular OCT scans (Optovue iVue 100; Optovue, Freemont, CA, USA) were performed by a trained member of staff: the 3D Macula protocol was used, comprising 128 B-scans over a 6 mm square centered on the fovea.^11^ Scans were taken from both eyes. Macular layer thicknesses were derived for ETDRS subfields using automated layer segmentation software (Orion; Voxeleron LLC, San Francisco, CA, USA). The layer chosen was the ganglion cell/inner plexiform layer. The nine ETDRS macular subfields conventionally used are as follows: a central subfield with a diameter of 1 mm; four subfields comprising the nasal, superior, temporal, and inferior parts of an inner annulus around the central subfield (the inner and outer diameters of the annulus are 1 and 3 mm, respectively); four subfields comprising the nasal, superior, temporal and inferior parts of an outer annulus (with inner and outer diameters of 3 and 6 mm). Figure 1 depicts the subfields; the nasal and temporal subfields were selected for the present study.

### Analyses

After exclusion of unreliable scans or segmentation data, data were included for complete twin pairs with known zygosity. The “inner ratio” was calculated by dividing the GCIPL thickness for the inner temporal subfield by the thickness for inner nasal subfield. The “outer ratio” was calculated using the corresponding thicknesses for the outer temporal and nasal subfields. The ratios were averaged for both eyes where data for both eyes were available (right and left eye ratios were not found to be significantly different). Participants with outlying values more than 4 standard deviations from the mean were excluded (with the assumption that this would be likely to relate to errors of segmentation or measurement, or to pathology). Scans from participants with conditions that could affect GCIPL measurements (such as glaucoma) or the reliability of segmentation (such as exudative AMD) were also excluded.

Distributions of inner and outer ratios were plotted (separately for Twin 1 and Twin 2 to check for consistency of findings). Average ratios were compared between participants of white and non-white ethnicity and between male and female participants. Coefficients of correlation with age were calculated for inner and outer ratios. For the age analysis, data were averaged for both members of each twin pair (since data within twin pairs are themselves correlated). Next, coefficients of intrapair correlation were calculated for MZ and DZ twins. Spearman coefficients were used as data were found to differ significantly from a normal distribution (Kolmogorov-Smirnov test). Age-adjusted heritability was estimated using maximum likelihood structural equation twin modelling as described previously,^5^ using the OpenMx package (https://openmx.ssri.psu.edu/), version 2.19, in the R statistical computing environment (http://www.r-project.org), version 4.1.1.

### Dataset From Second Imaging Platform

A smaller cohort of participants (less than 200) underwent macular OCT imaging with a different platform (3D OCT; Topcon Corporation, Tokyo, Japan) as part of a previous study.^5^ GCIPL thicknesses for inner and outer nasal and temporal subfields were extracted using the same automated segmentation software (Orion, version 3.1.0003a, Academic Build Aug-04-2021; Voxeleron LLC), and inner and outer ratios were calculated. Mean values were compared with the main cohort, and correlation with age was examined.

## Results

### Cohort Demographics, Mean Ratios, Sex Differences, and Correlation With Age

**Figure 2.**
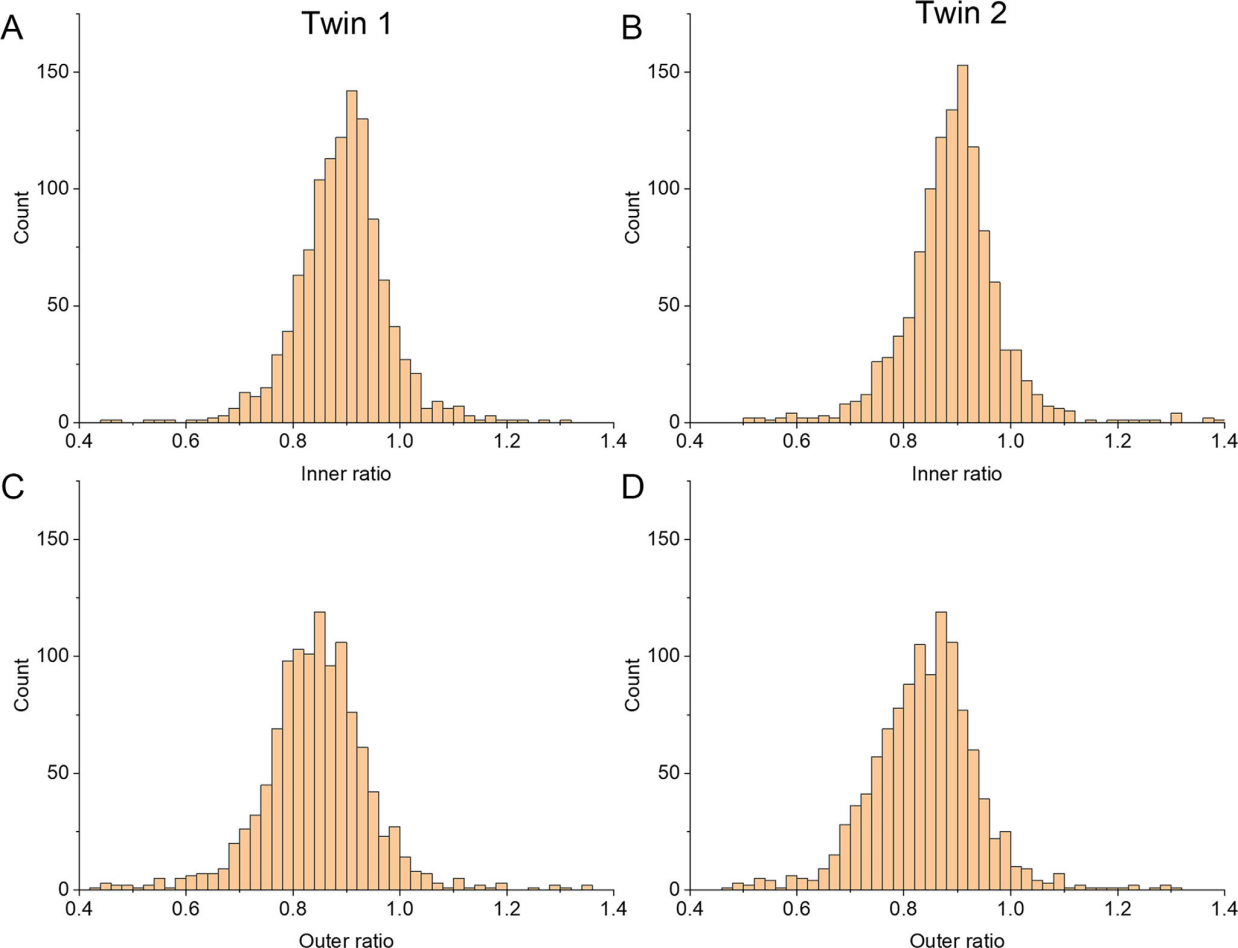
Histograms showing distributions of inner and outer ratios. Inner ratio distributions for Twin 1 (**A**) and Twin 2 (**B**). Outer ratio distributions for Twin 1 (**C**) and Twin 2 (**D**). The designations of Twin 1 and Twin 2 are essentially random (relating to which participant was recruited first), and the finding of similar distributions for both groups (and the apparent shifting of the distribution of outer ratios to lower values in both cases) suggests these findings are internally reproducible.

Data from 2300 twins were included in the main study. This included 739 MZ pairs and 411 DZ pairs. Mean (SD) age was 53.9 (16.5) years; 87% were female and 95% were white European (reflecting the overall demographics of the TwinsUK cohort). The mean (SD) inner and outer ratios were 0.89 (0.09) and 0.84 (0.11), respectively. The mean outer ratio was significantly lower than the mean inner ratio (*P* < 0.00001). Figure 2 shows distributions of inner and outer ratios, plotted separately for Twin 1 and Twin 2. The distributions for outer ratios are shifted toward lower values compared with inner ratios, and this is seen both for Twin 1 and Twin 2.

Table 1 gives summary statistics for the inner and outer ratios, including 2.5th and 97.5th centile values. When grouping participants by sex, the mean ages for females (n = 2000) and males (n = 300) were similar: mean (SD) ages were 54.1 (16.3) years and 52.9 (17.8) years (*P* = 0.27). Both inner and outer ratios were significantly higher in males compared with females (*P* = 0.0038 and 3.5 × 10^−^^9^ respectively). Summary statistics for male and female participants are given in Table 1. Of participants with recorded ethnicity (n = 2282), there was no significant difference between average ratios of white (n = 2174) and non-white (n = 108, comprising 18 Asian, 56 Black, and 34 “mixed” or “other”) participants when adjusted for age: *P* values were 0.58 and 0.32 for inner and outer ratios, respectively.

**Table 1.** Summary Statistics for Inner and Outer Ratios for the Main Cohort (n = 2300) and Separately for Female (n = 2000) and Male (n = 300) Participants

	Inner Ratio			Outer Ratio		
Parameter	All	Females	Males	All	Females	Males
Mean	0.89	0.89	0.90	0.84	0.84	0.88
Standard deviation	0.09	0.09	0.08	0.11	0.11	0.11
Median	0.90	0.90	0.90	0.84	0.84	0.87
2.5^th^ Centile	0.71	0.70	0.78	0.61	0.61	0.69
97.5^th^ Centile	1.07	1.07	1.09	1.05	1.04	1.16
Correlation with age (Spearman)	−0.17	−0.19	0.002	−0.21	−0.22	−0.13)
	(*P* = 1 × 10^−8^)	(*P* = 1 × 10^−9^)	(*P* = 0.98)	(*P* = 2 × 10^−13^)	(*P* = 9 × 10^−13^)	(*P* = 0.10)
Change per decade (standard error)	−0.0066 (0.0012)	−0.0079 (0.0013)	NS	−0.0097 (0.0015)	−0.0105 (0.0016)	NS

OCTs were obtained with the Optovue device (Optovue iVue 100, Optovue, Freemont, CA, USA) and segmented with automated software (Orion, Voxeleron LLC). For correlation with age and change per decade, the values from both twins from each twin pair were averaged. For male participants, no significant association with age was observed. NS, not significant.

**Figure 3.**
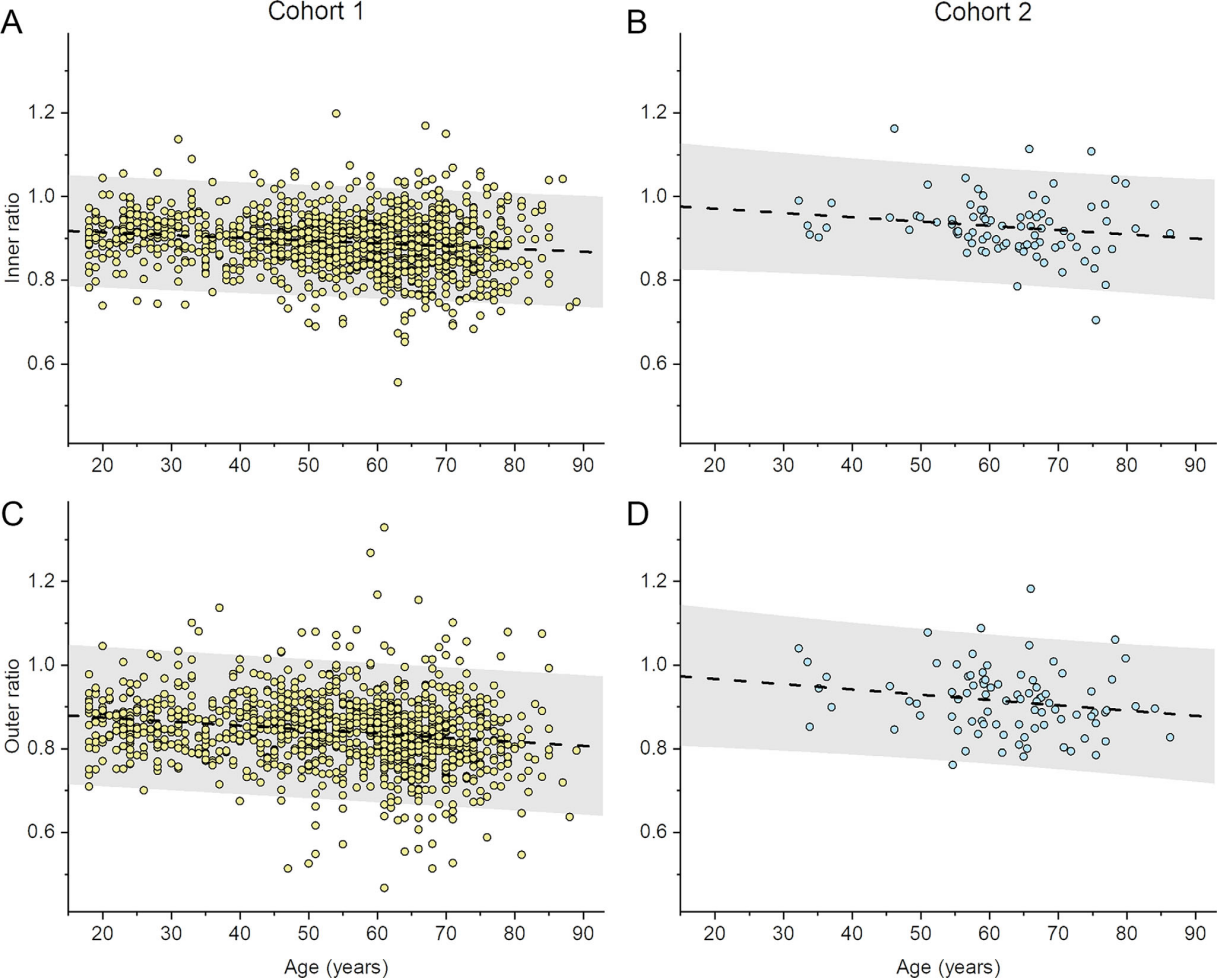
Ratios plotted against age for the main cohort (*left-hand* *panels*) and the second cohort (*right-hand panels*). The ratios have been averaged for both twins in each pair. The dashed lines show simple linear fits. The gray-shaded area denotes the 95% prediction band (where a future observation has 95% probability of being within this area). (**A**) Inner ratios for main cohort. Equation for linear fit: *y* = 0.927 – 0.000659*x*. For the upper and lower limits of the prediction band, linear fits have y-intercepts of 1.060 and 0.795 respectively and slopes of −0.000659. (**B**) Inner ratios for second cohort. Equation for linear fit: *y* = 0.991 – 0.00102*x*. For the upper and lower limits of the prediction band, linear fits have y-intercepts of 1.138 and 0.845 respectively and slopes of −0.00112 and −0.000926, respectively. (**C**) Outer ratios for main cohort. Equation for linear fit: *y* = 0.894 – 0.000968*x*. For the upper and lower limits of the prediction band, linear fits have y-intercepts of 1.060 and 0.727, respectively and slopes of −0.000968. (**D**) Outer ratios for second cohort. Equation for linear fit: *y* = 0.992 – 0.00126*x*. For the upper and lower limits of the prediction band, linear fits have y-intercepts of 1.156 and 0.828 respectively and slopes of −0.00137 and −0.00115, respectively.

The left-hand panels of Figure 3 plot inner and outer ratios against age (upper and lower panels, respectively), with the dashed black lines representing simple linear fits. The gray-shaded area represents the 95% prediction band (a future observation would have 95% probability of falling within this area). Equations of linear fits are given in the figure legend. A weak but highly significant negative correlation was observed with age for both inner and outer ratios, with correlation coefficients of −0.17 and −0.21, respectively. Linear fits gave losses of 0.0066 and 0.0097 per decade in the inner and outer ratios, respectively. Right-hand panels of Figure 3 show data from the second cohort, which will be described later.

**Figure 4.**
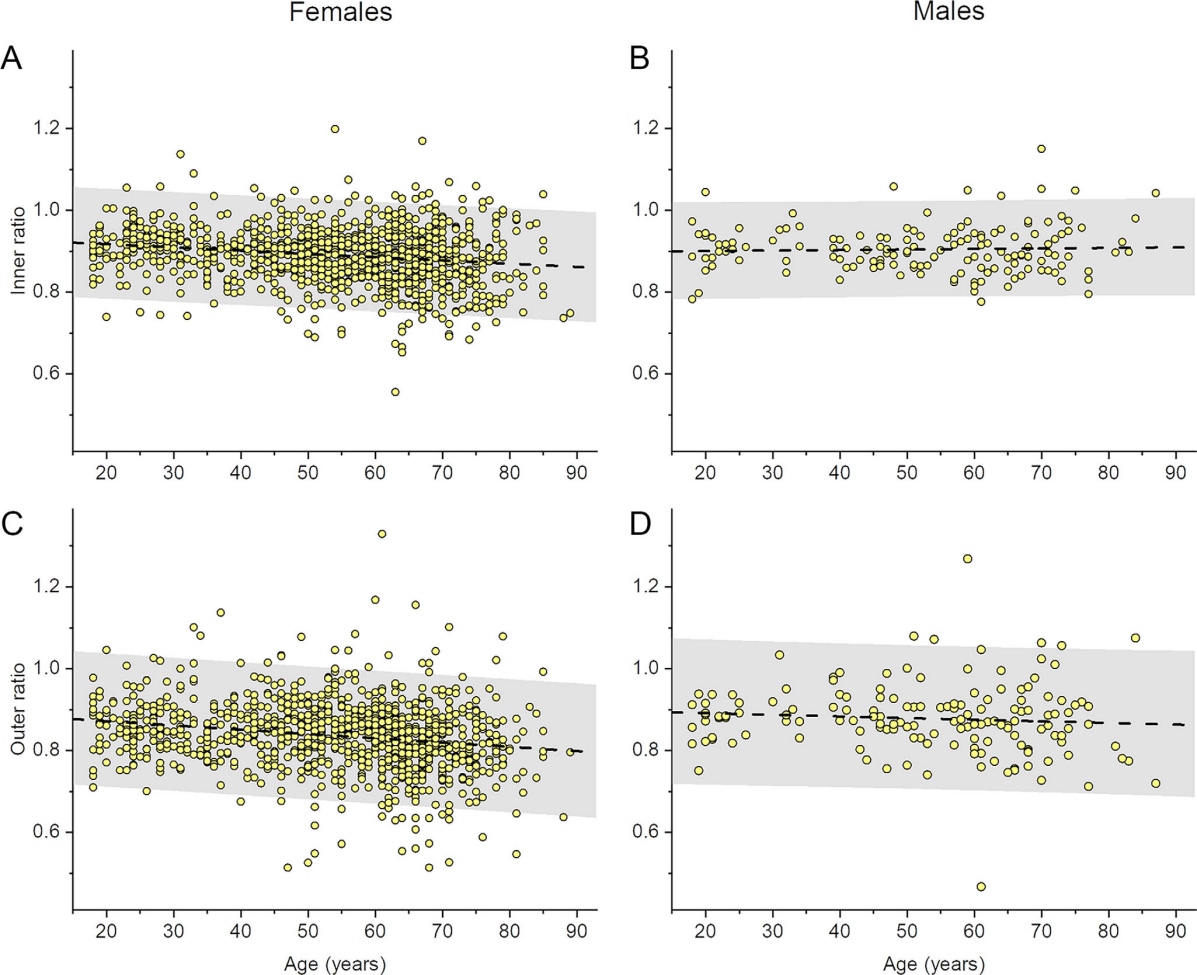
Ratios plotted against age for females (*left-hand* *panels*) and the males (*right-hand panels*) from the main cohort. The ratios have been averaged for both twins in each pair. The *dashed lines* show simple linear fits. The gray-shaded area denotes the 95% prediction band (where a future observation has 95% probability of being within this area). (**A**) Inner ratios for females (1000 twin pairs). Equation for linear fit: *y* = 0.933 – 0.000792*x*. For the upper and lower limits of the prediction band, linear fits have y-intercepts of 1.067 and 0.798 respectively and slopes of −0.000792. (**B**) Inner ratios for males (150 twin pairs). Equation for linear fit: *y* = 0.898 + 0.000133*x*. For the upper and lower limits of the prediction band, linear fits have y-intercepts of 1.015 and 0.780 respectively and slopes of 0.000135 and 0.000130 respectively. The slope with age for males was not significantly different from zero. (**C**) Outer ratios for females. Equation for linear fit: *y* = 0.893 – 0.00105*x*. For the upper and lower limits of the prediction band, linear fits have y-intercepts of 1.055 and 0.730 respectively and slopes of −0.00105. (**D**) Outer ratios for males. Equation for linear fit: *y* = 0.899 – 0.000399*x*. For the upper and lower limits of the prediction band, linear fits have y-intercepts of 1.076 and 0.722 respectively and slopes of −0.000395 and −0.000403, respectively. The slope with age for males was not significantly different from zero.

Figure 4 plots inner and outer ratios separately for females and males. In females, a significant negative correlation was observed with age for inner and outer ratios, with a simple linear fit giving a loss of 0.0079 per decade. Interestingly for males (right panels), no significant correlation with age was observed, and the slope of the linear regression was not significantly different from zero for both inner and outer ratios.

### Intrapair Correlations and Heritability

**Figure 5.**
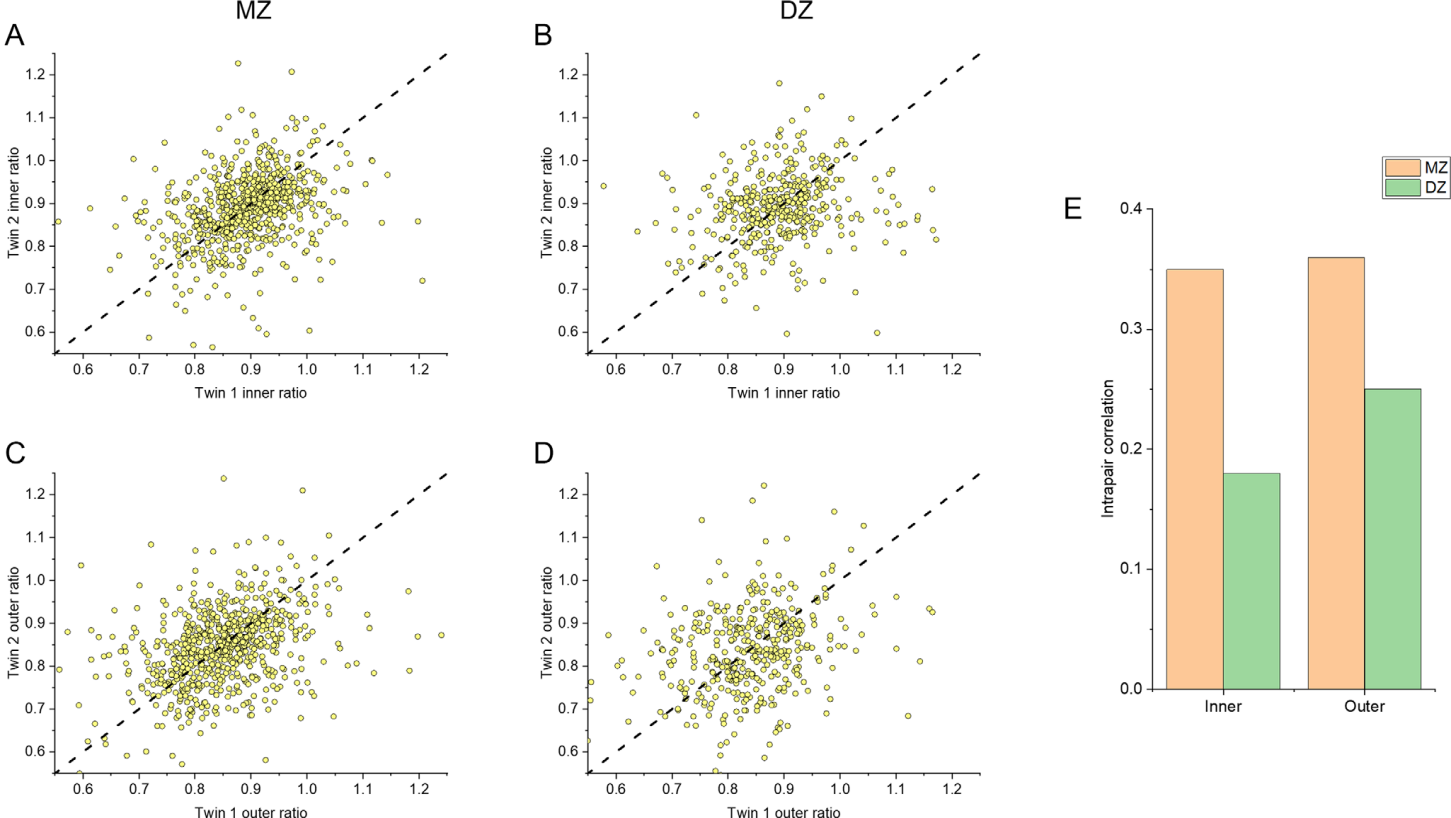
Ratios for Twin 2 plotted against Twin 1 for each pair and coefficients of correlation. (**A**) Inner ratios plotted for MZ pairs. (**B**) Inner ratios plotted for DZ pairs. (**C**) Outer ratios plotted for MZ pairs. (**D**) Outer ratios plotted for MZ pairs. (**E**) Coefficients of intrapair correlation for MZ and DZ pairs.

Figure 5 presents ratios for Twin 2 plotted against Twin 1 in each pair for MZ and DZ twins in the main cohort. The dashed 45-degree lines denote identical ratios for both twins. Although there is some scatter, ratios for MZ twins are more tightly clustered around the dashed line than those for DZ twins. Panel E compares coefficients of intrapair correlation: for inner ratios, MZ and DZ intrapair correlation coefficients were 0.35 and 0.18 respectively; for outer ratios, coefficients were 0.36 and 0.25, respectively. All intrapair correlations coefficients were significant (*P* < 0.001).

For heritability estimation, the most parsimonious model to explain the variance in the data for both inner and outer ratios was the AE model, where additive genetic factors (A) and unique environmental factors (E) contribute. The age-adjusted estimates of heritability (with 95% confidence interval) thus derived were 0.20 (0.14–0.26) and 0.23 (0.16–0 .29) for inner and outer ratios, respectively.

### Second Cohort Imaged With Different OCT Platform

For the second cohort, data were included from 166 twin participants. Mean (SD) age was 62.0 (11.5) years. Table 2 gives the summary statistics from this cohort. Mean (SD) inner and outer ratios were 0.93 (0.08) and 0.91 (0.09), respectively. A weak negative correlation with age was again observed. The difference between mean inner and outer ratios fell short of significance (*P* = 0.076). When compared with the ratios obtained from the Optovue images (larger cohort), those obtained from the Topcon images were significantly larger (*P* < 0.0001 for both inner and outer ratio comparisons), despite the greater average age of the latter cohort. Inner and outer ratios from the second cohort were plotted against age in the right-hand panels of Figure 3. A similar negative relationship with age is seen in both cohorts, though the absolute ratios are higher on average for the second cohort across the age range. Because there were very few males (fewer than 10) in the second cohort, a comparison between sexes was not performed.

**Table 2.** Summary Statistics For Inner And Outer Ratios for the Second Cohort

Parameter	Inner Ratio	Outer Ratio
Mean	0.93	0.91
Standard deviation	0.08	0.09
Median	0.93	0.91
2.5th Centile	0.75	0.76
97.5th Centile	1.09	1.07
Correlation with age (Spearman)	−0.19	−0.19
	(*P* = 0.073)	(*P* = 0.080)
Change per decade (standard error)	−0.010	−0.013
	(0.007)	(0.007)

OCTs were obtained with the Topcon device (3D OCT; Topcon Corporation) and segmented with automated software (Orion; Voxeleron LLC). For correlation with age and change per decade, the values from both twins from each twin pair were averaged.

## Discussion

This study explored ratios of temporal to nasal macular GCIPL thicknesses (from both the inner and outer ETDRS subfields) in a large healthy adult twin cohort. These ratios have potential clinical utility in aiding diagnosis of conditions such as albinism, which has been associated with smaller ratios. We present distributions for these ratios in healthy individuals, giving 2.5th and 97.5th centiles that could serve as a reference range. We found a weak, but significant negative correlation with age, and also found significant heritability. We found that males had higher ratios on average than females, and that, interestingly, no significant correlation was found with age for males. We also found that when these ratios were obtained from segmentations of scans obtained using a different device, the absolute values of the ratio were significantly different; nevertheless, a similar weak negative correlation with age was seen.

Relationships between retinal layer thicknesses and age have been explored in several studies.^5^^,^^12^^–^^18^ Most have reported a reduction in ganglion cell layer and IPL thickness in older age groups. In the present study, we found a weak but highly significant relationship such that older participants had lower ratios on average; this was seen for both inner and outer ratios and for both OCT devices (although the relationship with age failed to reach significance for the outer ratio with the Topcon device; this was likely to be related to the much smaller sample size for this cohort). If the absolute reduction with age were similar for nasal and temporal subfields, then mathematically (given the temporal subfield GCIPL is thinner than the nasal subfield GCIPL in most individuals), one would predict a reduction in the ratio with age. Thus a fall in the ratio with age does not necessarily indicate greater absolute age-related thinning in the temporal subfield.

The finding of slightly, but significantly, higher ratios in males might also relate to greater absolute retinal layer thicknesses in males compared with females, as reported in some studies. However, reasons for a sex-difference in the relationship with age are unclear. There were fewer male participants, and it is possible that, with a greater sample size, a significant correlation with age might emerge. Future studies examining relationships of this ratio with age in each sex would be informative.

Intrapair correlation was consistently greater for MZ than for DZ pairs for both the inner and outer ratios, indicating that genetic factors are likely to be significant (MZ pairs share all of their genes whereas DZ pairs share approximately half; the shared environment, however, is assumed to be similar for both MZ and DZ pairs). Our previous study confirmed significant heritability of segmented macular layer thicknesses,^5^ and in the present study we find significant heritability also for the inner and outer temporal to nasal GCIPL thickness ratios. Our point estimates of heritability were 0.20 and 0.23 for inner and outer ratios respectively. Thus, whilst rare genetic variants (such as those associated with albinism) may have large effects on the ratio, it appears that genetic factors also determine part of the variance of the ratio in healthy adults (albeit a small fraction). Future studies can explore the identity of these genetic factors, as well as the environmental factors that appear also to influence these ratios. From this study, one cannot identify the specific genetic or environmental factors; importantly, any measurement errors (which could relate to factors including scan centration or segmentation) would contribute to the environmental factors that appear to account for up to 77% to 80% of the variability.

Many studies have shown that retinal layer thicknesses can differ depending on the OCT device used.^18^^–^^22^ In the present study, we find that this also applies to ratios. Mean temporal to nasal macular subfield GCIPL ratios were significantly greater for scans taken with the Topcon 3D-OCT device compared with the Optovue device (for both inner and outer ratios), with both imaging datasets having been segmented by the same software. The findings thus highlight the importance of taking into account the device used when defining reference ranges or when comparing measurements between individuals or within the same individual at different time points. The reason for the differences is unclear. If there were consistent absolute, rather than relative, differences between layer thicknesses obtained with different devices, this could yield a difference in ratios (as discussed in terms of age above). We did not find noticeable differences between scan quality by visual inspection. Future studies could explore differences in scan and segmentation quality more quantitatively.

Inevitably, there are limitations to the present study that merit consideration. We investigated the combined GCIPL layer (rather than only the ganglion cell layer) as given by the segmentation algorithm; separate ganglion cell layer and IPL thicknesses were not available. Although both ganglion cell and IPL topography are altered in patients with albinism, it has been demonstrated that parsing these layers is likely to yield greater information.^8^ In future, separate segmentation of these layers in our twin cohort would allow heritability of each layer and temporal-to-macular ratios to be assessed separately.

Other limitations include the specific demographics of the TwinsUK cohort, which might limit generalizability to other cohorts with different age, sex and ethnic characteristics. TwinsUK was initially set up to investigate osteoporosis and osteoarthritis (conditions that are more prevalent in women) and was later widened to investigate a range of other conditions; the strong female preponderance has persisted. The correlation with age might not hold for age groups outside the specific age range. Many patients with suspected albinism are assessed at younger ages, and it is unclear whether the age-relationship could be extrapolated to younger ages. A linear fit was used, but the true relationship may not necessarily be linear. The study was also cross-sectional; a longitudinal study would be needed to more reliably investigate the effect of age within individuals. The comparison by ethnicity is likely to be limited by the much smaller numbers of non-white participants. The overall findings, particularly the absolute values, may also be specific to the OCT devices used and the specific segmentation technique. Axial lengths and other factors can also influence layer thicknesses, but these data were not available for the majority of the cohort. Axial length differences between participants would mean that the actual ETDRS subfield sizes would differ (although if defined in angular terms, then the sizes would be more consistent).

In summary, our study highlights the range of temporal to nasal macular subfield GCIPL ratios in a large cohort, identifying correlations with age, sex differences, and significant heritability, as well as showing similarities and differences in findings when different OCT platforms are used. Future studies could investigate other OCT devices and segmentation algorithms and seek to identify the specific genetic and environmental factors affecting spatial distribution of macular ganglion cells in healthy individuals, as well as exploring factors that might modify the extent of foveal hypoplasia seen in albinism.
